# Tetris Genioplasty: A New Paradigm for Chin Asymmetries Correction

**DOI:** 10.3390/jcm12237354

**Published:** 2023-11-28

**Authors:** Valerio Ramieri, Linda Latini, Guido Gabriele, Vittoria Fantozzi, Tito Matteo Marianetti, Flavia Cascino

**Affiliations:** 1 Ortognatica Roma, Via Nomentana 311, 00137 Roma, Italy; valerioramieri@gmail.com (V.R.); info@myrhinoplasty.it (T.M.M.); 2 Maxillofacial Surgery Unit, Department of Mental Health and Sense Organs, Azienda Ospedaliera Universitaria Senese, 53100 Siena, Italy; guido.gabriele@unisi.it (G.G.); vitto.fanto@gmail.com (V.F.); flaviacascino@hotmail.com (F.C.)

**Keywords:** asymmetry surgery, chin genioplasty, genioplasty, orthognathic surgery

## Abstract

The chin plays a crucial role as a fundamental structural component that contributes to the overall aesthetics and harmony of the face. Recognizing its central position, medical science has seen the evolution of numerous surgical techniques over the years, all aimed at correcting the range of structural irregularities that can affect the chin. In this contribution, the authors introduce an innovative osteotomy technique, aimed at cases of chin asymmetry in which the skeletal median diverges from the dental median. This technique, called “Tetris genioplasty”, involves performing the classic rectangular osteotomy, but includes an additional vertical osteotomy in order to obtain two distinct segments. Finally, these segments are translocated and repositioned to obtain a realignment between the skeletal median and the dental median. The results were entirely satisfactory for the patients, aligning perfectly with the expected appearance after the operation. Furthermore, no complications were reported, proving the success and safety of the procedure. The Tetris genioplasty aligns itself with this progressive trend by offering a minimally invasive method that nevertheless is able to achieve excellent results with a high impact on the patient’s quality of life, presenting a promising path in the pursuit of optimal aesthetic results with minimized patient morbidity and greater overall safety.

## 1. Introduction

The chin represents one of the most important structures for facial harmony and aesthetics, determining the attractiveness of the profile. Its anatomy can be very variable, and its characteristics are sometimes a distinctive feature of the person, or sometimes a pathological element to be treated surgically [1].

According to Naini et al. [2], a retrusion or protrusion of up to 4 mm is basically imperceptible, while surgery is desirable for protrusions of more than 6 mm and for retrusions of more than 10 mm. Over the years, the aesthetic parameters defining the ideal chin have changed a great deal, and today patients’ desires do not always meet the canons of beauty that can be traced back to the neoclassical canons of Latin and Greek art [3]. For example, the search for chin characteristics that for years were true gender markers, e.g., a higher, wider, and often more projecting chin in men and a softer, more tapered, or oval-looking chin in women [4,5], has been declining in recent years due to a progressive masculinization of female aesthetic standards.

This change in ideal references plays an important role in diagnosis and treatment planning. Over the years, various techniques have been developed to treat various abnormalities of the chin, from the placement of prostheses made of alloplastic material to various osteotomy techniques [6,7,8].

Alterations in size and shape must be considered as an excess or deficiency of hard or soft tissue in one of three planes: anteroposterior, vertical, or transverse. For an anterior-only increase or reduction in the chin, the osteotomy must be performed in a horizontal plane. However, by changing the angle of the osteotomy, the vertical dimension will be affected by moving the genial segment up or down. In addition, chin height can be further controlled by osteotomy of a genial bone segment or augmentation by hair grafting. Transverse problems that produce chin asymmetry with mismatched facial, mental, and dental midlines can be corrected with a horizontally sliding genioplasty [9,10].

The technique of choice for the individual patient must be studied according to the abnormality reproduced and the desired result [11].

Recent years have seen an increase in demand for the corrective surgery of facial asymmetries, in which chin assessment plays an important role [12]. In fact, chin asymmetries represent a real challenge for the maxillofacial surgeon who must study the most effective techniques to guarantee a good aesthetic result [13].

This is not always possible with current genioplasty techniques. Recently, more and more patients are facing compensatory orthodontic treatments that result in the centering of the dental midline on asymmetrical faces. Therefore, in cases where the midline of the teeth does not correspond to the skeletal midline, moving the tip of the chin laterally has limitations. The alignment of the facial midline may cause a discontinuity of the outer edge of the mandible that is not always acceptable to the patient [14].

In this manuscript, we have described the “Tetris genioplasty”, an osteotomy designed to correct these types of chin asymmetries. This technique provides a minimally invasive approach with few operative risks.

## 2. Material and Method

One of the authors performed five Tetris genioplasties in 2022, all of which were included in this case series. Inclusion criteria included asymmetry of the chin in the frontal plane, with misalignment of the skeletal midline relative to the dental midline, and patients undergoing previous orthodontic camouflage treatment. Exclusion criteria were patients undergoing different types of genioplasty. Genioplasty combined with a bimaxillary procedure was not an exclusion criterion.

All patients complained only of a cosmetic deficit before the operation. None complained of a functional disorder, such as a disturbance in breathing, chewing, swallowing, or phonation.

Table 1 summarizes the patients’ data.

All patients underwent a preoperative axial thin-cut (0.6 mm) Cone-Beam Computed Tomography (CBCT) scan. The data were recorded in a generic Digital Imaging and Communications in Medicine (DICOM) format and transferred to Dolphin Imaging Software 12.0, a software dedicated to orthognathic Virtual Surgical Planning (VSP). The software reformats the DICOM images into 3D STL files [15]. Patients were photographed before surgery and at each postoperative visit from frontal, oblique, basal, and lateral views.

All patients signed informed consent according to the principles of the Declaration of Helsinki and signed consent for the publication of their data and photographs.

Ethical approval was sought from the Institutional Review Board (IRB) of the University of Siena, and the approval number is 9/2021.

The entire procedure was performed under general anesthesia with nasotracheal intubation; about 10 min before the incision, plexiform anesthesia was performed to achieve hemostasis. The incision was made with an electric scalpel approximately 15 mm from the mucogingival line. The mental muscle was dissected subperiosteally, so exposure of the mental nerves and excessive dissection of the area are unnecessary. According to the telescopic genioplasty [16], the osteotomy has a rectangular shape, the transverse limit is determined by the intercanthal distance, and the height should be between 15 and 20 mm from the mandibular border, considering the roots of the lower incisors.

**Figure 1:**
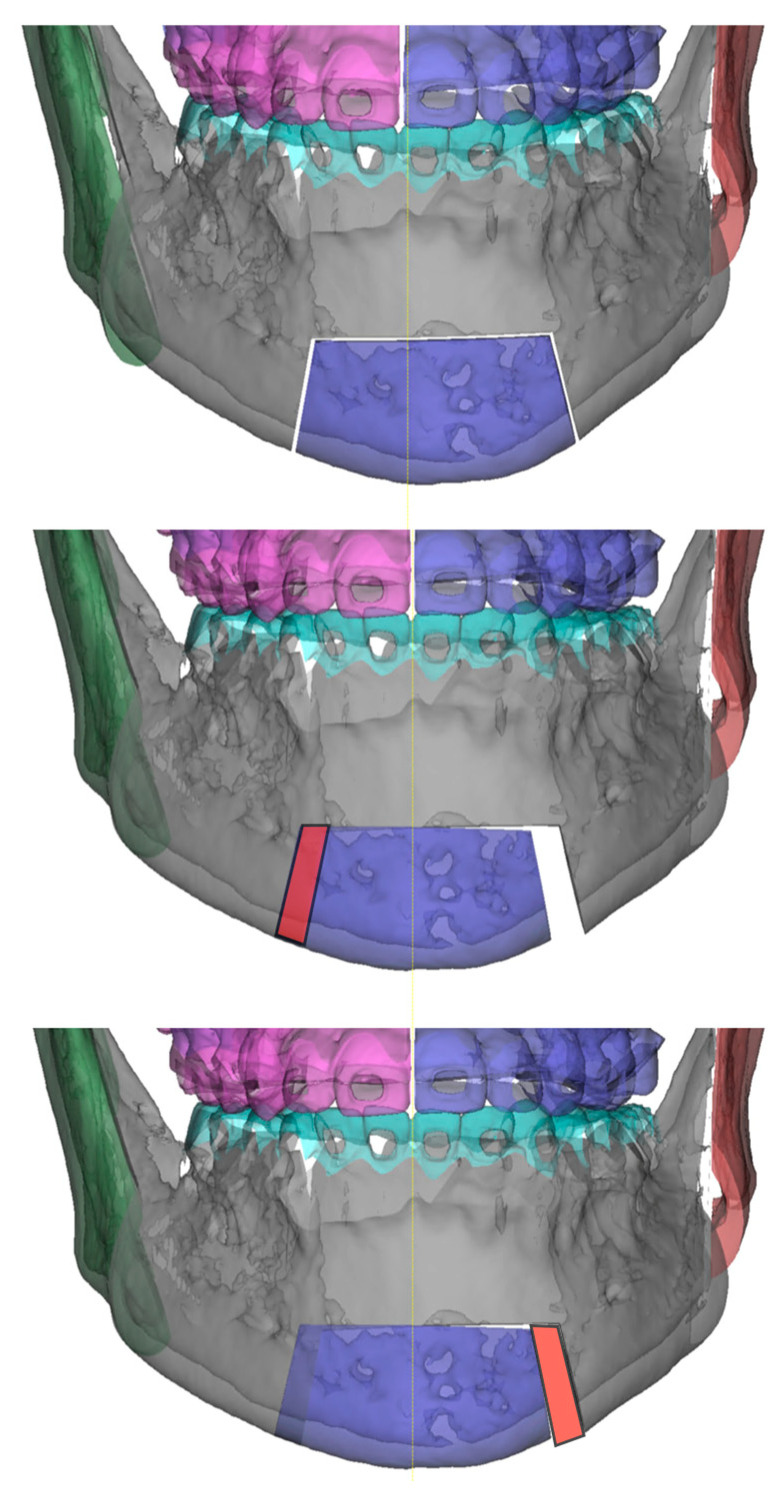
Surgical planning.

In telescopic genioplasty, a single rectangular block is cut and mobilized, while in Tetris genioplasty, the osteotomy design includes an additional vertical osteotomy. The width of this segment must be digitally assessed to match the skeletal midline with the dental midline (Figure 1).

**Figure 2:**
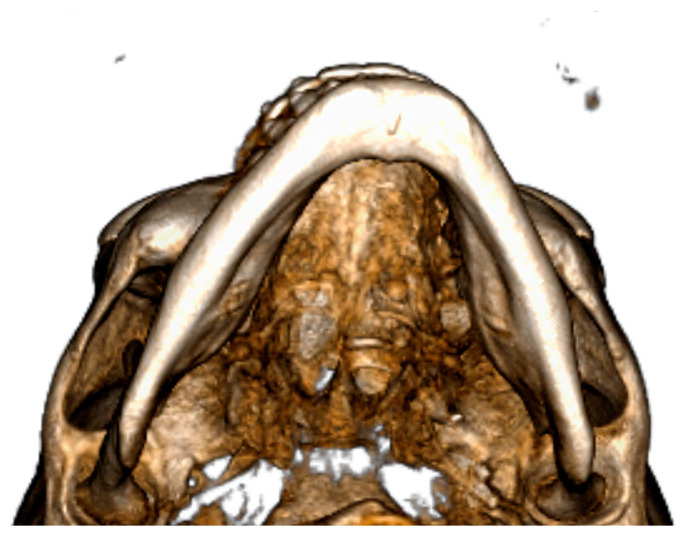
Detail of genioglossus insertion.

During digital planning, another referral point used for the real skeletal midline is the insertion of the genioglossus on the interior surface of the mandible (Figure 2).

**Figure 3:**
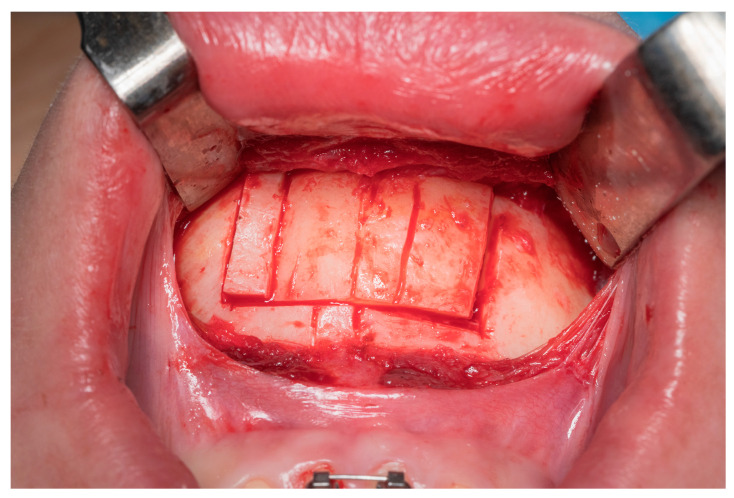
Description of vertical line from left to right: left osteotomy, skeletal midline, dental midline, Tetris osteotomy, and right osteotomy.

**Figure 4:**
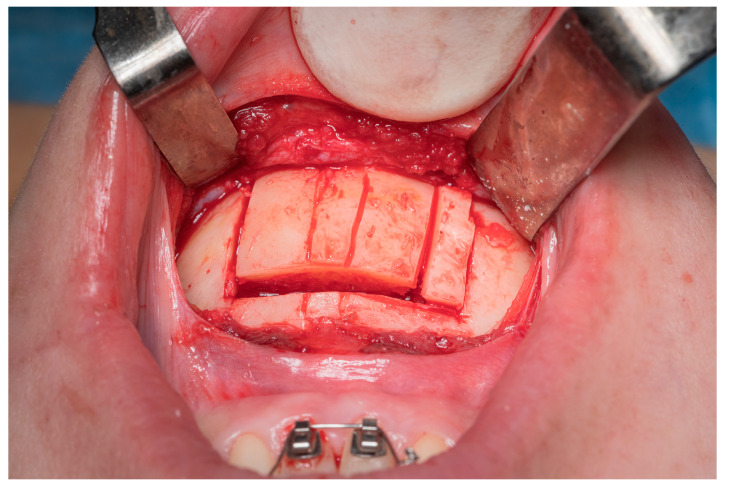
Intraoperative view of Tetris block reposition.

The empty space is then filled by translating the chin and matching the midline. The newly created void is then filled with the previously removed bone block on the opposite side (Figure 3 and Figure 4). Osteotomies are fixed with mini plates and screws.

The operation was followed by a 24 h hospital stay in cases of isolated genioplasty, while a two-night hospital stay was necessary for cases where the genioplasty was combined with a bimaxillary procedure.

Postoperatively, an electronic pulp tester was used to check the viability of the lower incisor. The average follow-up time was 6 months, with a range of 3 to 8 months. In most cases, the follow-up consisted of a clinical examination 1 week after surgery, then again 3 months after surgery, and then again after 6 months. All follow-ups were tailored to the needs of the individual patient, being able to end earlier in the case of a patient in excellent clinical condition, or being extended in the case of a patient with additional needs.

## 3. Results

Table 1 shows the measurement of the width of the deviation between the skeletal and dental median in each patient.

No postoperative complications such as infection, plate or screw extrusion, non-union, and chin asymmetry were observed during the follow-up period. No change in the vitality of the lower anterior teeth was detected [17].

All patients found the result satisfactory and in line with pre-operative expectations. As this is an aesthetic procedure and the patient did not complain of any functional impairment, we believe that subjective facial analysis is an excellent parameter to consider.

**Figure 5:**
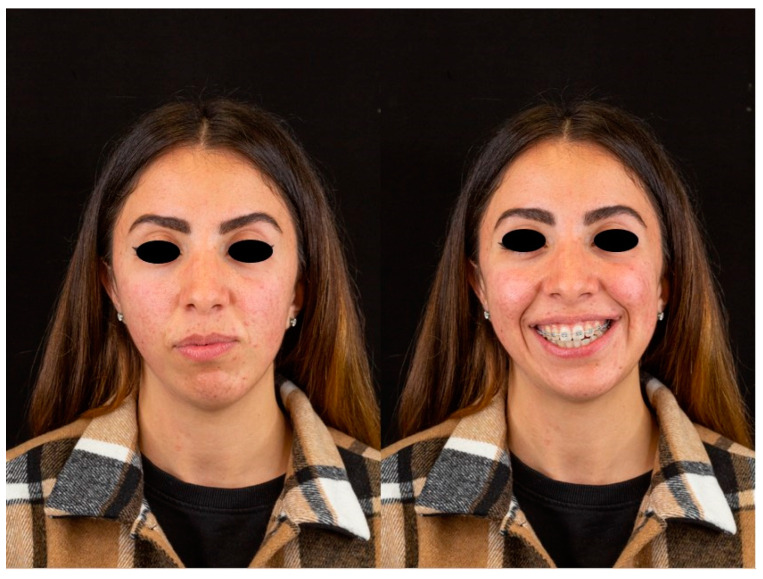
Pre-operative appearance.

**Figure 6:**
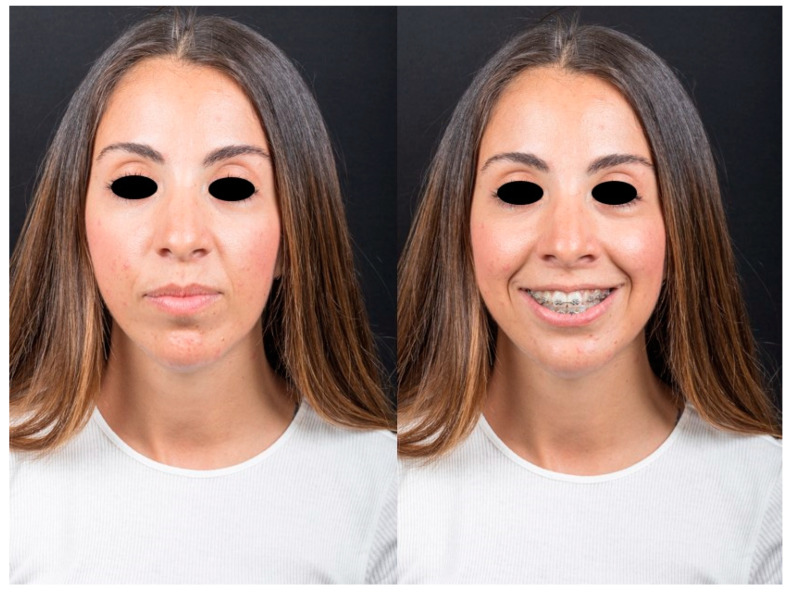
Post-operative appearance.

A patient’s pre-operative and post-operative appearance are shown in Figure 5 and Figure 6, respectively.

## 4. Discussion

The chin, an integral component of the human face, occupies a position of primary importance in facial harmony and aesthetics. Its features significantly influence the perception of an individual’s overall appearance, and its profile plays a crucial role in determining attractiveness. However, the chin is not a unique feature: its anatomy presents a considerable degree of variability between individuals. In some cases, these distinct attributes of the chin give a person a unique identity, while in others they may present themselves as pathological elements that require surgery for correction.

The research conducted by Naini et al. delves into the nuances of chin protrusion and retrusion, shedding light on the perceptual thresholds that guide the decision-making process for surgical treatment. The results suggest that minimal alterations of up to 4 mm in either direction—protrusion or retrusion—often go unnoticed. However, the consensus for surgical intervention emerges when protrusion exceeds 6 mm or retrusion exceeds 10 mm. This benchmark provides valuable information, helping surgeons in their evaluation and recommendations for patients wishing to undergo chin surgery.

The evolution of society’s beauty standards has significantly influenced the perception of an ideal chin in different eras. Historical standards of beauty, particularly those inspired by the neoclassical principles of Latin and Greek art, outlined the parameters for an aesthetically pleasing chin. Over the years, these standards have shifted and transformed in response to cultural changes, artistic reinterpretations, and changing preferences. As a result, the notion of an ‘ideal’ chin is no longer rigidly bound to historical conventions. Contemporary ideals are influenced by a dynamic interplay between cultural norms, media representations, and individualistic expressions of beauty.

An intriguing aspect of this evolution lies in the change in gender-specific attributes that have long characterized chin aesthetics. Traditionally, chin characteristics were used as gender markers: men often displayed taller, wider, and more prominent chins, while women tended towards softer, tapered, or oval shapes. However, changes in society, including the redefinition of gender norms and a broader spectrum of gender identities, have led to a reassessment of these standards. The feminization of masculine traits and the masculinization of feminine traits have introduced a new level of complexity to chin aesthetics. As a result, the dichotomous distinctions that once characterized chin aesthetics are giving way to a more fluid and personalized approach.

In the wake of the global pandemic, an unprecedented surge in the use of web-based meeting platforms has emerged, marking a pivotal moment in human history. Unlike any previous case, our collective dependence on these platforms has increased exponentially. Interestingly, the construct of the brain has been inherently shaped by mirror images. However, these software interfaces present users with the perspective of an outside observer. This marked departure from the norm allows for the cultivation of a new perceptual experience for a large portion of the population. As a result, there has been a noticeable surge in the desire for facial asymmetry correction, a phenomenon likely catalyzed by abrupt and prolonged exposure to this unique virtual viewpoint [18,19].

Faced with this scenario, the surgeon is faced with a complex decision-making process. With the imperative to achieve optimal results and address even the most subtle irregularities, one must choose from a range of surgical techniques, sometimes requiring the exploration of new strategies.

In the context of medical practice, these evolving ideals have tangible implications for diagnosis and treatment planning. Divergence from traditional gender-based standards requires a nuanced understanding of each patient’s unique desires and aspirations. Medical professionals must engage patients in comprehensive discussions, addressing their aesthetic goals and considering the evolving parameters of chin aesthetics. Adapting treatment plans to these changing ideals and individual preferences underscores the importance of a patient-centered approach in modern medical practice.

Over the years, a variety of techniques and interventions have been developed to address the various irregularities of the chin. These interventions range from the placement of alloplastic materials such as chin implants to complex osteotomy procedures that involve cutting and repositioning bone. The choice of the most appropriate technique depends on a meticulous analysis of the specific chin abnormality and the desired outcome. This underscores the individualized nature of modern medical interventions, in which each case is treated as unique and requires a tailored approach to achieve optimal results.

The increased demand for corrective surgeries to correct facial asymmetries has further accentuated the importance of chin assessment. Facial asymmetry, often due to developmental or congenital factors, presents a complex challenge for maxillofacial surgeons. In these cases, the role of the chin goes beyond its individual aesthetics and is intertwined with overall facial balance. Achieving symmetry in the presence of asymmetry requires a deep understanding of both the artistic principles of facial aesthetics and the technical nuances of surgery.

However, as medical practice evolves, we also see the complexity of patient cases. The current landscape of facial aesthetics and corrective procedures reveals an increasing emphasis on holistic treatments. Compensatory orthodontic interventions, designed to align dental midlines in the context of facial asymmetry, have introduced new dimensions to the role of the chin in the treatment paradigm. This has led to a transformation in the approach to surgical corrections. Moving the tip of the chin laterally to align it with the midline of the face does not always produce the desired results. The interaction between dental and skeletal midlines presents challenges that surgeons must address with precision and innovation.

In cases of asymmetry where the dental midline deviates from the skeletal midline, conventional osteotomy approaches may prove ineffective. Despite efforts to move the chin laterally to align it with the facial midline, correction of the outer edge remains a challenge, making classical techniques inadequate. This underscores the need for innovative solutions to address these intricate anatomical nuances and achieve successful aesthetic results.

In response to these challenges, the “Tetris genioplasty” technique has emerged as a promising innovation in the correction of chin asymmetry.

Motivated by the work of Nelson et al. on telescopic genioplasty [16], the authors embarked on a creative trajectory to introduce an innovative osteotomy called “Tetris genioplasty”. This nomenclature draws a playful parallel with the iconic video game, failing to highlight its operational similarity. This technique intricately addresses chin asymmetry like the interlocking shapes of the game. The term encapsulates the innovative essence of the surgical approach, cleverly alluding to its methodology through a familiar and understandable reference.

This technique is very useful because it provides an optimal result through a minimally invasive approach, allowing complex asymmetries to be corrected.

This technique offers the advantage of avoiding the need for prostheses made of alloplastic material. Although these prostheses may be useful in selected cases, their use may increase patient morbidity and carry a non-negligible risk of infection [20,21]. This technique, which avoids the use of such prostheses, improves patient outcomes by reducing potential complications, consistent with a patient-centered approach to care.

As in all osteotomies, in “Tetris genioplasty”, it is important to consider that by performing the cuts with both Piezosurgery and the saw, a very small portion of bone tissue will be lost, so the final juxtaposition of all bone segments will always leave a small vertical defect due to the thickness of the instrument adopted.

This could be a hindrance to the technique, but the authors believe it is sufficient for the surgeon to be aware of this aspect to calculate it and design the osteotomy by taking it into consideration. Also, in cases where genioplasty is combined with orthognathic surgery, small bone chips can be taken from the other surgical sites to fill these gaps [22,23].

Meticulously addressing the complexities of facial midline alignment, the “Tetris genioplasty” technique aims to provide a minimally invasive yet effective solution. Its focus on preserving aesthetic results and reducing operative risks underscores the continuing quest for advances in surgical approaches.

In conclusion, the importance of the chin transcends its anatomical boundaries. It serves as a canvas on which the evolution of beauty standards, gender aesthetics, and surgical innovations is vividly painted. As societal ideals change and medical practices advance, the chin remains a nexus of art and science, where the principles of aesthetics are harmoniously intertwined with the precision of surgical techniques. In an age characterized by individuality and inclusiveness, the chin is a testament to the ever-evolving nature of human perception and the remarkable ability of medicine to adapt, innovate, and improve lives.

## 5. Conclusions

Through the application of the “Tetris genioplasty” technique, the authors achieved remarkable aesthetic results, demonstrating its effectiveness in correcting complex asymmetries of the chin that had not previously been resolved using alternative osteotomy methods. This approach has proven particularly suitable for resolving asymmetries occurring in the frontal plane.

Despite some inherent technical challenges, the authors firmly believe that the minimally invasive nature of this technique has a significant level of efficacy, especially in scenarios where the dental midline deviates significantly from the skeletal midline. The ability of the technique to address this specific subset of asymmetries underscores its nuanced utility. By succeeding in resolving asymmetries that have eluded correction by conventional osteotomies, “Tetris genioplasty” demonstrates its ability to produce aesthetically satisfactory results. Although every surgical technique involves some technical complexities, the authors’ confidence in the efficacy of the procedure underscores the potential of overcoming these challenges.

The attractiveness of the technique lies in the ability to correct asymmetries without the use of implants, thus mitigating the potential complications associated with alloplastic materials. As surgical procedures continue to evolve, the emphasis on minimally invasive approaches becomes increasingly pronounced. “Tetris genioplasty”, in this context, aligns with this progressive trend, offering a method that achieves results while minimizing invasiveness.

Its ability to achieve favorable results where other methods fail reflects its potential to revolutionize chin aesthetics.

## Figures and Tables

**Table 1:** Patient Data.

	Age	Sex	Impairment Complained	Previous Orthodontic Treatment	Surgery Procedure	Deviation between Skeletal Median and Dental Median
Patient 1	28	Female	Aesthetic Impairment	Yes	Bimaxillary Procedure + Tetris genioplasty	3.17 mm
Patient 2	23	Female	Aesthetic Impairment	Yes	Tetris genioplasty	2.84 mm
Patient 3	31	Female	Aesthetic Impairment	Yes	Bimaxillary Procedure + Tetris genioplasty	3 mm
Patient 4	29	Female	Aesthetic Impairment	Yes	Bimaxillary Procedure + Tetris genioplasty	3.77 mm
Patient 5	40	Female	Aesthetic Impairment	yes	Tetris genioplasty	2.47 mm

## Data Availability

Data are contained within the article.

## References

[1] Nguyen J.D., Duong H. (2023). Anatomy, Head and Neck: Face. StatPearls.

[2] Naini F.B., Donaldson A.N., McDonald F., Cobourne M.T. (2012). Assessing the influence of chin prominence on perceived attractiveness in the orthognathic patient, clinician and layperson. Int. J. Oral Maxillofac. Surg..

[3] Bueller H. (2018). Ideal Facial Relationships and Goals. Facial Plast. Surg..

[4] Deschamps-Braly J. (2019). Feminization of the Chin: Genioplasty Using Osteotomies. Facial Plast. Surg. Clin. N. Am..

[5] Gulati A., Knott P.D., Seth R. (2022). Sex-Related Characteristics of the Face. Otolaryngol. Clin. N. Am..

[6] Ferretti C., Reyneke J.P. (2016). Genioplasty. Atlas Oral Maxillofac. Surg. Clin. N. Am..

[7] Ramieri V., Vellone V., Marianetti S., Marianetti T.M. (2021). Captain America’s Shield Genioplasty. J. Craniofac. Surg..

[8] Ramieri V., Maffìa F., Vellone V., Marianetti S., Marianetti T.M. (2021). The Pyramid Chin Augmentation: A New Technique. J. Craniofac. Surg..

[9] Japatti S.R., Chourasia N., Siddegowda C.Y., Shriram P., Yajurvedi R., Beawerwala T. (2020). Sagittal Genioplasty: New Techniques. J. Maxillofac. Oral Surg..

[10] Ramanathan M., Panneerselvam E., Parameswaran A., Kanno T. (2023). Genioplasty in Contemporary Orthognathic Surgery. Oral Maxillofac. Surg. Clin. N. Am..

[11] Maglitto F., Sani L., Piloni S., Del Prete G.D., Arena A., Committeri U., Salzano G., Califano L., Friscia M. (2022). Step-technique genioplasty: A case report. Int. J. Surg. Case Rep..

[12] Kim H.J., Noh H.K., Park H.S. (2023). Nonsurgical orthodontic correction of facial asymmetry by condylar remodeling and mandibular repositioning following occlusal cant correction with microimplants: A case report. Angle Orthod..

[13] Dong T., Ye N., Yuan L., Wu S., Xia L., Fang B. (2020). Assessing the Influence of Chin Asymmetry on Perceived Facial Esthetics With 3-Dimensional Images. J. Oral Maxillofac. Surg..

[14] Agrawal M., Agrawal J.A., Nanjannawar L., Fulari S., Kagi V. (2015). Dentofacial Asymmetries: Challenging Diagnosis and Treatment Planning. J. Int. Oral Health.

[15] Gennaro P., Chisci G., Aboh I.V., Gabriele G., Cascino F., Iannetti G. (2014). Comparative study in orthognathic surgery between Dolphin Imaging software and manual prediction. J. Craniofac. Surg..

[16] León N.J., Pereira Pérez A.J., Requejo S., Gòmez D., Mendes Barros H.L. (2021). Telescopic genioplasty: A new concept to reshape the chin. Adv. Oral Maxillofac. Surg..

[17] Gennaro P., Giovannoni M.E., Pini N., Aboh I.V., Gabriele G., Iannetti G., Cascino F. (2017). Relationship Between the Quantity of Nerve Exposure During Bilateral Sagittal Split Osteotomy Surgery and Sensitive Recovery. J. Craniofac. Surg..

[18] Bianchi I., Savardi U. (2008). The relationship perceived between the real body and the mirror image. Perception.

[19] Özgür E., Muluk N.B., Cingi C. (2017). Is Selfie a New Cause of Increasing Rhinoplasties?. Facial Plast. Surg..

[20] Agnihotry A., Fedorowicz Z., Nasser M., Gill K.S. (2017). Resorbable versus titanium plates for orthognathic surgery. Cochrane Database Syst. Rev..

[21] Van Camp P., Verstraete L., Van Loon B., Scheerlinck J., Nout E. (2021). Antibiotics in orthognathic surgery: A retrospective analysis and identification of risk factors for postoperative infection. Int. J. Oral Maxillofac. Surg..

[22] McGuire C., Boudreau C., Prabhu N., Hong P., Bezuhly M. (2022). Piezosurgery versus Conventional Cutting Techniques in Craniofacial Surgery: A Systematic Review and Meta-Analysis. Plast. Reconstr. Surg..

[23] Cascino F., Aboh I.V., Giovannoni M.E., Pini N., Zerini F., Del Frate R., Carangelo B.R., Xu J., Gabriele G., Gennaro P. (2021). Orthognathic surgery: A randomized study comparing Piezosurgery and Saw techniques. Ann. Ital. Chir..

